# Fownes’ Manual of Chemistry

**Published:** 1886-01

**Authors:** 


**Fownes' Manual of Chemistry—Theoretical and Practical.** New American from the Twelfth English edition, embodying "Watts' Physical and Inorganic Chemistry," with 168 illustrations. Philadelphia: Lea Brothers & Co. 1885.

There are some books which perpetually renew their youth, and this is certainly one of them. The writer well remembers poring over Fownes during his first year at college, now twenty years ago. Since that time, many editions of this work have appeared, and each one has marked the steady and wonderful progress which has been made in chemical knowledge. Although

Professor Fownes died many years ago, the editors of the work have succeeded in keeping it in the van among all rivals, and the present edition will sustain its reputation as one of the best books on the subject in the language.

The publishers have issued it in a most attractive style; although it contains more than one thousand pages, it is still convenient to handle, and the original arrangement, which has proved so convenient, is, as far as possible, retained.

